# Discovery of gene-alcohol interaction loci influencing blood pressure in 1.1 million individuals from multiple populations

**DOI:** 10.21203/rs.3.rs-9283196/v1

**Published:** 2026-04-03

**Authors:** Mary Feitosa, Karen Schwander, Clint Miller, Aldi Kraja, Amy Bentley, Michael Brown, Hannah de Hesselle, Raymond Noordam, Songmi Lee, Pavithra Nagarajan, Heming Wang, Ayush Giri, Farrah Ammous, Traci Bartz, Chiara Batini, Jean-Tristan Brandenburg, Max Breyer, Heather Cordell, Janie Corley, Latchezar Dimotrov, Anh Do, Jiawen Du, Franco Giulianini, Christopher Grace, Valborg Gudmundsdottir, Xiuqing Guo, Sarah Harris, Natalie Hasbani, Janina Herold, Keiko Hikino, Edith Hofer, Andrea Horimoto, Fang-Chi Hsu, Zhijie Huang, Anne Jackson, Chang Hoon Kang, Federica Laguzzi, Timo Lakka, Christophe Lefevre, Jian’an Luan, Leo-Pekka Lyytikäinen, Aline Meirhaeghe, Manon Muntaner, Masahiro Nakatochi, Giuseppe Giovanni Nardone, Ilja Nolte, Teresa Nutile, Nicholette Palmer, Amit Patki, Alessandro Pecori, Varun Rao, Anne Richmond, Mercedes Richter, Mihir Sanghvi, Aurora Santin, Heather Stringham, Fumihiko Takeuchi, Ye An Tan, Jingxian Tang, Maris Teder-Laving, Olga Trofimova, Stella Trompet, Peter van der Most, Ya Xing Wang, Zhe Wang, Yujie Wang, Wenyi Wang, Erin Ware, Stefan Weiss, Kenneth Westerman, Chenglong Yu, Wanying Zhu, Md Abu Yusuf Ansari, Pramod Anugu, Anna Argoty-Pantoja, John Attia, Bernhard Banas, Lydia Bazzano, Joshua Bis, Carsten Boger, Jennifer Brody, Ulrich Broeckel, Harry Campbell, Archie Campbell, Pasqualina Cennamo, William Checkley, Miao-Li Chee, Guanjie Chen, Yii-Der Chen, Kayesha Coley, Stacey Collins, Jean Dallongeville, Hithanadura Janaka de Silva, Charles Dupont, Todd Edwards, Christian Enzinger, Jessica Faul, Lilian Fernandes Silva, Adam Gepner, Anuj Goel, Mathias Gorski, Mariaelisa Graff, C. Charles Gu, Jiang He, Sami Heikkinen, Erin Hill-Burns, Adriana Hung, Steven Hunt, Marguerite Irvin, Mika Kähönen, Sharon Kardia, Minjung Kho, Heikki Koistinen, Ivana Kolčić, Pirjo Komulainen, José Eduardo Krieger, Lenore Launer, Daniel Levy, Jianjun Liu, Joseph McCormick, John McNeil, yuri milaneschi, Jaime Miranda, Kari North, Anniina Oravilahti, Alison Pattie, Patricia Peyser, Giulia Pianigiani, Leslie Raffel, Olli Raitakari, Michele Ramsay, Salil Redkar, Paul Redmond, Paul Ridker, Frits Rosendaal, Daniela Ruggiero, Tom Russ, Charumathi Sabanayagam, Alyssa Scartozzi, Reinhold Schmidt, Laura Scott, Rodney Scott, Susan Shenkin, Roelof Smit, Jennifer A. Smith, Zhi Da Soh, Beatrice Spedicati, David Stott, Quan Sun, Gerald Sze, E Shyong Tai, Paola Tesolin, Rima Triatin, Nataraja Sarma Vaitinadin, Rob van Dam, Julien Vaucher, Uwe Völker, Henry Völzke, Chaolong Wang, Helen Warren, Rajitha Wickremasinghe, Ko Willems van Dijk, Cancan Xue, Ken Yamamoto, Jie Yao, Mitsuhiro Yokota, Martina Zimmermann, Philippe Amouyel, Jennifer Below, Sven Bergmann, Antonio Bernabe-Ortiz, Michael Boehnke, Palwende Boua, LifeLines Cohort study, Donald Bowden, Daniel Chasman, Ching-Yu Cheng, Marina Ciullo, Maria Pina Concas, Simon R. Cox, Luc Dauchet, Ian Deary, Stephan Felix, Ervin Fox, Nora Franceschini, Barry Freedman, Paolo Gasparini, Giorgia Girotto, Vilmundur Gudnason, Caroline Hayward, Iris Heid, Elizabeth Holliday, Sahoko Ichihara, Catherine John, Jost Jonas, J Wouter Jukema, Mart Kals, Norihiro Kato, Bernard Keavney, Tanika Kelly, Markku Laakso, Paul Lacaze, Leslie Lange, Karin Leander, Seunggeun Lee, Terho Lehtimäki, Changwei Li, Ching-Ti Liu, Ruth Loos, brenda penninx, Alexandre Pereira, Ozren Polasek, Bruce Psaty, Rainer Rauramaa, Charles Rotimi, Jerome Rotter, Igor Rudan, Helena Schmidt, Xueling Sim, Harold Snieder, Klaus Stark, Chikashi Terao, Lynne Wagenknecht, Nicholas Wareham, Hugh Watkins, David Weir, Kristin Young, Wei Zhao, William Gauderman, Alanna Morrison, Myriam Fornage, Han Chen, Jeffrey O’Connell, Alisa Manning, Paul de Vries, Lisa de las Fuentes, Dabeeru Rao, Patricia Munroe, Michael Province, Thomas Winkler

**Affiliations:** Washington School of Medicine; Washington University; University of Virginia; University of Mississippi Medical Center; National Institutes of Health, NHGRI; The University of Texas Health Science Center at Houston; University of Regensburg; Leiden University Medical Center; University of Texas Health Science Center at Houston, McGovern Medical School; Brigham and Women’s Hospital; Brigham and Women’s Hospital; Harvard Medical School; Vanderbilt University Medical Center; University of Michigan; University of Washington; University of Leicester; Vanderbilt University School of Medicine; Newcastle University; University of Edinburgh; Wake Forest University School of Medicine; Washington University School of Medicine, St. Louis, MO, USA; Brigham and Women’s Hospital; Radcliffe Department of Medicine, University of Oxford; Faculty of Medicine, University of Iceland; Lothian Birth Cohorts, Dept. Psychology; The University of Texas Health Science Center at Houston; University of Regensburg; RIKEN; Medical University Graz; Brigham and Women’s Hospital; Wake Forest School of Medicine; Tulane University School of Public Health and Tropical Medicine; Department of Biostatistics and Center for Statistical Genetics, School of Public Health, University of Michigan, Ann Arbor, MI; Seoul National University; 11. Institute of Environmental Medicine, Karolinska Institute, Stockholm; Institute of Biomedicine/Physiology, University of Eastern Finland, Kuopio; University of Newcastle; University of Cambridge; Fimlab Laboratories and Finnish Cardiovascular Research Center; Inserm; University of Lille, Inserm, Institut Pasteur de Lille; Nagoya University Graduate School of Medicine; Institute for Maternal and Child Health—IRCCS; University of Groningen, University Medical Center Groningen; Institute of Genetics and Biophysics “A. Buzzati-Traverso”, CNR; Department of Genetics and Genomic Sciences, Icahn School of Medicine at Mount Sinai; University of Alabama at Birmingham; Institute for Maternal and Child Health - IRCCS, Burlo Garofolo; University of Maryland; The University of Edinburgh; University of Regensburg, University Hospital Regensburg; Queen Mary University of London; University of Trieste; University of Michigan; Japan Institute for Health Security; National University of Singapore and National University Health System, Singapore; Boston University; University of Tartu; University of Lausanne; Leiden University Medical Center; University Medical Center Groningen; Tsinghua University; University of Alabama at Birmingham; Leiden University Medical Center; University of Michigan; University Medicine Greifswald & University of Greifswald; Massachusetts General Hospital; Monash University; Vanderbilt University Medical Center; University of Mississippi Medical Center; University of Mississippi Medical Center; University of Groningen, University Medical Center Groningen; University Hospital Regensburg; Tulane University; University of Washington; Universitätsklinikum Regensburg; University of Washington; Medical College of Wisconsin; University of Edinburgh; Institute of Genetics and Biophysics ‘A. Buzzati-Traverso’, CNR; Johns Hopkins University; Singapore Eye Research Institute, Singapore National Eye Centre, Singapore; National Human Genome Research Institute; Harbor-UCLA Medical Center; University of Leicester; University of Michigan; Lille University Hospital; University of Kelaniya; Vanderbilt University Medical Center; Department of Veterans Affairs; Medical University Graz; University of Michigan; University of Eastern Finland; University of Wisconsin School of Medicine and Public Health; Radcliffe Department of Medicine, University of Oxford; University of Regensburg; University of North Carolina at Chapel Hill; Washington University School of Medicine; University of Texas Southwestern Medical Center; Institute of Biomedicine, School of Medicine, University of Eastern Finland, Kuopio Campus; University of Michigan; VA Tennessee Valley Healthcare System; University of Utah School of Medicine; University of Alabama at Birmingham School of Public Health; Tampere University; University of Michigan; Seoul National University; University of Helsinki, Helsinki University Hospital, Finnish Institute for Health and Welfare; University of Split; Kuopio Research Institute of Exercise Medicine, Kuopio, Finland; University of Sao Paulo, Laboratory of Genetics and Molecular Cardiology; National Institute on Aging, National Institutes of Health; National Heart, Lung, and Blood Institute, National Institutes of Health; Genome Institute of Singapore; The University of Texas Health Science Center at Houston (UTHealth) School of Public Health; Monash University; Amsterdam UMC, Vrije Universiteit/GGZ inGeest; The University of Sydney; Department of Epidemiology, University of North Carolina at Chapel Hill, Chapel Hill, NC; University of Eastern Finland; Department of Epidemiology, School of Public Health, University of Michigan; Institute for Maternal and Child Health - IRCCS “Burlo Garofolo”; University of California Irvine; Turku University Hospital and Research Centre of Applied and Preventive Cardiovascular Medicine, University of Turku; University of the Witwatersrand; Washington University School of Medicine; Edinburgh University; Leiden University Medical Center; Institute of Genetics and Biophysics “A. Buzzati-Traverso”, CNR; University of Edinburgh; Singapore Eye Research Institute; Vanderbilt University School of Medicine; Medical University of Graz; University of Michigan; University of Newcastle and the Hunter Medical Research Institute; University of Edinburgh; University of Copenhagen; Department of Epidemiology, School of Public Health, University of Michigan, Ann Arbor, MI, USA; Singapore Eye Research Institute; University of Trieste/Institute for Maternal and Child Health, I.R.C.C.S. “Burlo Garofolo”; University of Glasgow; Children’s Hospital of Philadelphia; University of Leicester; Saw Swee Hock School of Public Health, National University of Singapore and National University Health System; University of Trieste; UMCG; Vanderbilt University Medical Center; George Washington University; Lausanne University Hospital and University of Lausanne; Department of Functional Genomics, Interfaculty Institute for Genetics and Functional Genomics, University Medicine Greifswald, Greifswald, Germany; University Medicine Greifswald; Huazhong University of Science and Technology, Tongji Medical College; William Harvey Research Institute, Barts and The London School of Medicine and Dentistry, Queen Mary University of London; University of Kelaniya; Leiden University Medical Center; Singapore Eye Research Institute; Kurume University School of Medicine; Institute for Translational Genomics and Population Sciences/The Lundquist Institute at Harbor-UCLA Medical Center; Kurume University School of Medicine; University of Regensburg; Univ. Lille, Inserm, CHU Lille, Institut Pasteur de Lille, LabEx DISTALZ - U1167-RID-AGE Facteurs de risque et déterminants moléculaires des maladies liées au vieillissement; VUMC; University of Lausanne; Universidad Peruana Cayetano Heredia; University of Michigan; Clinical Research Unit of Nanoro, Institut de Recherche en Sciences de la Santé; Lifelines; Wake Forest School of Medicine; Brigham and Women’s Hospital; National University of Singapore; Istitute of Genetics and Biophysics A. Buzzati-Traverso - CNR; Institute for Maternal and Child Health - IRCCS; Lothian Birth Cohorts, Department of Psychology, University of Edinburgh; Lille University Hospital; University of Edinburgh; Department of Internal Medicine B (Cardiology); University of Mississippi Medical Center; University of North Carolina; Wake Forest University School of Medicine; IRCCS-Burlo Garofolo / University of Trieste; Institute for Maternal and Child Health - IRCCS “Burlo Garofolo”; Icelandic Heart Association; Department of Genetic Epidemiology, University of Regensburg; Hunter Medical Research Institute; 28. Department of Environmental and Preventive Medicine, Jichi Medical University School of Medicine; University of Leicester; Rothschild Foundation Hospital, Institut Français de Myopie; Leiden University Medical Center; University of Tartu; National Center for Global Health and Medicine; Division of Cardiovascular Sciences, Faculty of Biology, Medicine and Health, University of Manchester,; University of Illinois at Chicago; University of Eastern Finland; Monash University; University of Colorado at Anschutz; 11. Institute of Environmental Medicine, Karolinska Institute, Stockholm; Seoul National University; Department of Clinical Chemistry, Fimlab Laboratories and Finnish Cardiovascular Research Center-Tampere, Faculty of Medicine and Health Technology, Tampere University; O’Donnell School of Public Health, UT Southwestern Medical Center; Boston University School of Public Health; University of Copenhagen; Amsterdam UMC; Massachusetts Veterans Epidemiology Research and Information Center (MAVERIC), VA Boston Healthcare System; Faculty of Medicine, University of Split; Cardiovascular Health Research Unit; Kuopio Research Institute of Exercise Medicine; National Institutes of health; The Lundquist Institute for Biomedical Innovation at Harbor-UCLA Medical Center; Centre for Global Health, Usher Institute, University of Edinburgh, Edinburgh, Scotland, UK; Gottfried Schatz Research Center (for Cell Signaling, Metabolism and Aging), Medical University of Graz, Austria; National University of Singapore; University Medical Center Groningen; University Regensburg; RIKEN Center for Integrative Medical Sciences; Wake Forest School of Medicine; University of Cambridge; Oxford University; University of Michigan; University of North Carolina at Chapel Hill; University of Michigan; University of Southern California; The University of Texas Health Science Center at Houston; The University of Texas Health Science Center at Houston; New York University; University of Maryland School of Medicine; Broad Institute; The University of Texas Health Science Center at Houston; Washington University School of Medicine; Washington University in St. Louis; Queen Mary University; Washington University; University Regensburg

## Abstract

Genetic predisposition and alcohol consumption are risk factors for increased blood pressure (BP), but their interactions influencing BP remain understudied. We conducted population-specific and cross-population meta-analyses of genome-wide gene-alcohol (GxAlc) interactions affecting BP in >1.1M individuals from multiple populations. We identified 46 GxAlc interaction loci for BP, including 21 from one-degree-of-freedom interaction tests (PGxAlc<5×10–8; or <0.05/Meff, Meff independent BP associations at P<10–5), and 25 from two-degree-of-freedom tests of main and interaction effects (PGxAlc<0.05/M2df, M2df independent 2df-associations at P2df<5×10–8), including 7 novel and 39 known BP loci. The 12q24 locus highlights the genetic effect of BRAP-rs11066001 on BP, being ~6 times larger in current drinkers than in non-drinkers. Gene prioritization with 46 GxAlc loci identified 15 genes with ≥3 lines of evidence (location, literature, druggability, functional/regulatory annotation, or pathway analyses). Several loci showed sex- and population-specific effects and revealed biological pathways of alcohol’s influence on BP, suggesting mechanisms underlying alcohol-induced hypertension.

## Introduction

The association between alcohol consumption and high blood pressure (BP) is well established; however, the biological mechanisms underlying alcohol-induced hypertension (HTN) remain largely unknown. Epidemiological and experimental studies have demonstrated a dose-dependent relationship between alcohol consumption and BP, supporting a causal role of alcohol in HTN risk [1–5]. Both HTN and high alcohol consumption contribute to increased morbidity and mortality. High BP is associated with heart disease, stroke, kidney disease, and peripheral artery disease [3, 6]. In a two-decade follow-up study of over 9.3 million South Korean and nearly 7,000 U.S. adults, nonoptimal BP (≥120/80 mmHg or treated with antihypertensive medication) was present in over 95% of South Korean and 94.3% of U.S. individuals who developed cardiovascular disease (CVD), making it the leading contributor to CVD in both cohorts [7]. In the United States, HTN accounts for nearly 75 percent of CVD deaths [8]. Moreover, alcohol remains the most widely used psychoactive substance worldwide and a major driver of global population disease burden [9]. Drinking large amounts of alcohol (e.g., >7 drinks per week for females or >14 drinks per week for males) [10] increases the risk of developing numerous alcohol-related medical conditions, such as arterial HTN, alcoholic cardiomyopathy, cirrhosis, and alcohol use disorder (AUD) [9–13].

Differences in sex and population of origin are important factors influencing the relationship between alcohol and HTN [2]. Worldwide, the prevalence of alcohol use is higher in males than in females, and these patterns have remained relatively stable over time [9]. Studies indicate that there are sex differences in various aspects of alcohol consumption. Females and males differ in how alcohol affects BP, likely due to physiological, hormonal, metabolic, and behavioral factors [2, 4]. Despite the fact that males are more frequently diagnosed with AUD, females tend to experience more severe health problems related to alcohol, such as liver disease, certain cancers, and heart issues [14]. Alcohol consumption also varies markedly across populations, with the highest per capita alcohol consumption observed in populations from Europe and the Americas [9]. In addition to environmental, cultural, and socio-economic factors, the differences in alcohol consumption and HTN across populations can be partially explained by genetic effects. Differences in allele frequencies, linkage disequilibrium (LD) structures, and gene expression can lead to varying effects of alcohol and BP levels across populations [2, 15–17], emphasizing how population-specific evolutionary history influences the genetic factors underlying these traits.

Genome-wide association studies (GWAS) have proven a powerful method for identifying genetic variants associated with various traits and diseases, including BP, HTN, and alcohol consumption. Overall, GWAS have identified over 3,800 single-nucleotide polymorphisms and 1,165 independent loci associated with BP and HTN, implicating thousands of genes in BP regulation [18, 19]. GWAS have also identified loci associated with alcohol consumption and AUD [20, 21], including a study reporting 496 loci (849 variants) associated with the number of drinks per week [22]. Additionally, Mendelian randomization analyses of over 371K individuals further support genetic evidence of a causal, risk-increasing effect of alcohol on the risks of HTN and coronary artery disease [1]. Notably, several genes involved in alcohol metabolism are also associated with BP, suggesting shared biological pathways [16, 18].

The genetic loci identified for BP explain less than 10% of BP variability [18], highlighting the importance of other unaccounted factors such as gene-environment interactions, including gene-alcohol consumption (GxAlc) [6]. Modeling GxAlc interaction can reveal genetic associations that manifest only under specific levels of alcohol exposure. However, few studies have explored the interactions between genetic variants and alcohol because of the large sample sizes required to achieve adequate statistical power. We previously examined GxAlc interactions on BP in approximately 570K individuals, and identified novel BP loci through GxAlc interactions analyses [16]. Here, we expand this work to identify GxAlc interaction loci influencing systolic BP (SBP), diastolic BP (DBP), and pulse pressure (PP) by conducting sex- and alcohol-specific analyses in more than 1.1 million individuals from multiple populations. To investigate interaction related to alcohol dose dependence, we evaluated four alcohol exposure contrasts: current drinkers (CURDRINK), heavy versus never drinkers (HEAVYvsNEVER), light versus never drinkers (LIGHTvsNEVER), and heavy versus light drinkers (HEAVYvsLIGHT). Given established sex differences in alcohol’s effects on BP, analyses were further stratified by sex. Finally, we performed comprehensive bioinformatic analyses, druggability assessments, and literature reviews to assess the functional relevance of identified variants and their roles in BP regulation, alcohol metabolism, and shared biological pathways.

## Results

### Overview of the GWIS meta-analyses

**Figure 1.**
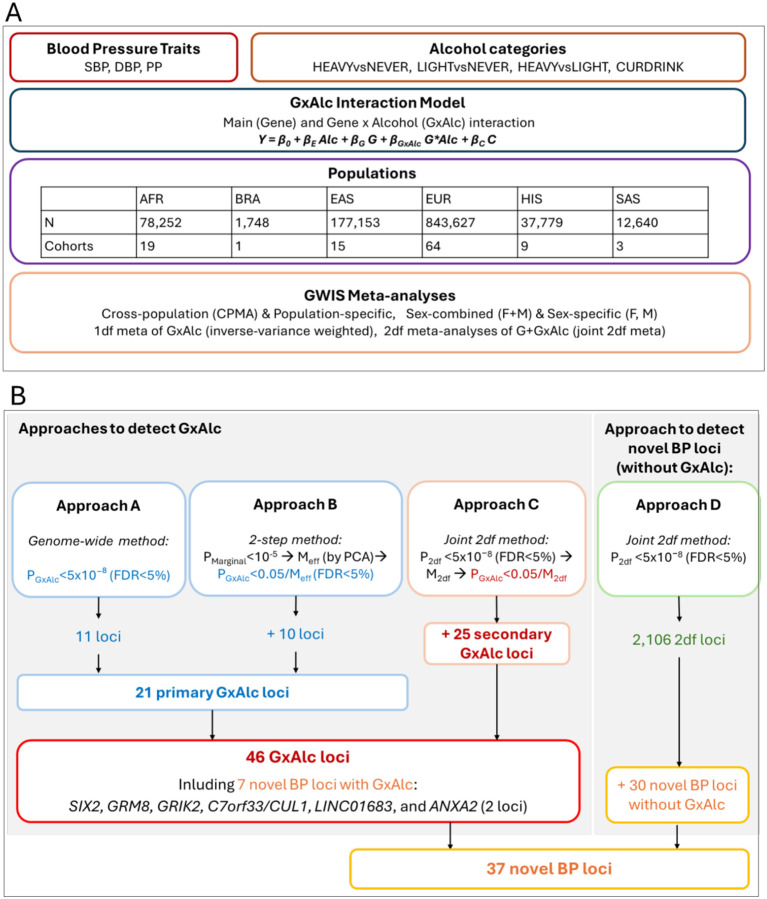
Workflow and overview of results. A. Overview of analyzed traits, alcohol exposures, sample sizes, and meta-analyses. B. Overview of approaches to detect GxAlc interaction and to detect novel BP loci, and of results by approach.

In a comprehensive **analysis**, 1,151,199 participants from 111 genome-wide interaction studies (GWIS) were included, representing multiple populations: 6.80% African (AFR), 0.15% Brazilian (BRA), 15.39% East Asian (EAS), 73.28% European (EUR), 3.28% Hispanic/Latino (HIS), and 1.10% South Asian (SAS) populations (**Supplementary Table 1, Supplementary Table 2**, Figure 1).

GxAlc interaction effects on BP traits (SBP, DBP, and PP) were modeled across each GWIS using alcohol exposure categories (CURDRINK, HEAVYvsNEVER, LIGHTvsNEVER, and HEAVYvsLIGHT) and sex groups (male, female, or combined) for each cohort (Methods). We performed inverse-variance weighted meta-analyses of GxAlc interaction effects (1df) [23]and two-degree-of-freedom (2df) joint main and interaction meta-analyses [24] using the summary-level statistics from the GWIS. These meta-analyses were conducted separately for the three BP traits, four alcohol exposure categories, and three sex groups, both within each population and as a cross-population meta-analysis (CPMA). The results identify BP loci exhibiting GxAlc interactions and uncover novel BP loci.

We present the results in a three-tiered approach (Figure 1). First, we searched for variants with GxAlc interaction on BP traits using a 1df test with two complementary methods. We examined GxAlc interaction at genome-wide significance among all variants (Approach *A*: P_GxAlc_<5×10^−8^ and FDR_GxAlc_<5%), and at Bonferroni-corrected significance among variants filtered for marginal association (Approach *B:* 2-step method; P_GxAlc_<0.05/M_eff_ and FDR_GxAlc_<5%; where M_eff_ is the number of effectively independent tests among variants with marginal P<1×10^−5^). Since the marginal association and the interaction tests are statistically independent, they can be applied consecutively within the same sample. Second, we searched for additional variants with GxAlc interaction on BP traits based on Bonferroni-corrected significance among variants with genome-wide significant 2df joint main and interaction effects (Approach *C*: P_GxAlc_<0.05/M_2df_; where M_2df_ is the number of loci with P_2df_<5×10^−8^ and FDR_2df_<5%). Importantly, the 2df joint test and the 1df GxAlc interaction test are statistically dependent. We thus consider loci identified by the 1df test (Approach *A*+*B*) as our primary GxAlc results, and those from the 2df joint test (Approach *C*) as secondary GxAlc results, which may require further confirmation. Third, we examined the 2df joint main and interaction meta-analysis results for additional novel BP loci without GxAlc interaction (Approach *D*: P_2df_<5×10^−8^, FDR_2df_<5%, and P_GxAlc_>0.05). Details of these criteria are provided in Methods and are illustrated in Figure 1.

### GWIS meta-analyses from 1df test identify primary 21 significant GxAlc interaction BP loci

We identified 21 primary independent genomic loci with significant GxAlc interactions from the 1df test (distance >500kb or r^2^<0.1 between loci; **Table 1, Supplementary Table 3, Supplementary Figures 1–3**). These include 11 loci with genome-wide significant GxAlc interactions (Approach *A*) and 10 additional loci identified only through the 2-step method (Approach *B*). Among the 21 GxAlc interaction loci, 3 have not been previously identified by other BP GWAS or GWIS (*LOC105370839/ANXA2*, *GRM8,* and *SIX2*).

**Figure 2:**
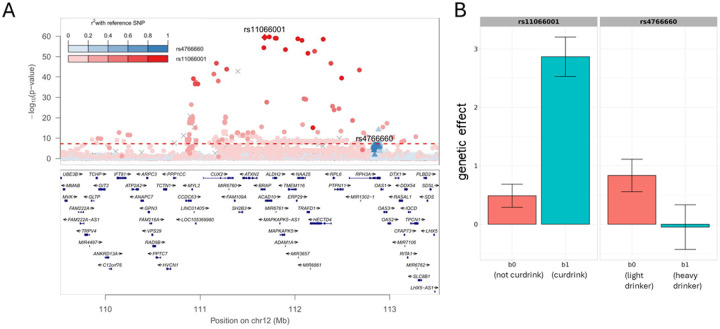
Two independent gene-alcohol interactions on 12q24, *BRAP/ALDH2* and *RPH3A*, in the EAS population. A. Regional association plot of GxAlc interaction for the *BRAP/ALDH2* (red) and *RPH3A* (blue) signals. B. Bar plots of subgroup-specific effect sizes at the two signals. Based on EAS-only meta-analyses, b_0_ and b_1_ are the estimated sex-combined genetic effect sizes on SBP in non-current drinkers (b_0_) and current drinkers (b_1_) for rs11066001, and male-specific effect sizes on SBP in light drinkers (b_0_) and heavy drinkers (b_1_) for rs4766660.

At the known genomic BP region on chromosome 12q24, two significant independent variants were identified based on LD (r^2^<0.1 and distance>1.1Mb, **Table 1**) at the *BRAP* and *RPH3A* genes. These loci were specific to the EAS population, being common in EAS and too rare to analyze in other populations (**Table 1**). The 12q24 region contained variants in genes associated with alcohol consumption in EAS, such as *ALDH2* [25–27], and variants in genes linked to oxidative stress response, including *BRAP* [28] (Figure 2A). Interactions at this EAS-specific locus were identified for SBP, DBP, and PP and across all alcohol exposures (CURDRINK, HEAVYvsNEVER, HEAVYvsLIGHT, and LIGHTvsNEVER). The *BRAP-*rs11066001 effect on BP was higher with alcohol consumption; for example, we observed a ~6-fold increase in genetic effects on SBP among current versus non-drinkers in both males and females (P_GxAlc_=3.5×10^−33^, Figure 2A, **Supplementary Figure 1**). Conversely, the other EAS-specific signal on 12q24, *RPH3A*-rs4766660, suggests that higher alcohol consumption (heavy versus light drinkers) reduced genetic effects on SBP in males (Figure 2B, **Supplementary Figure 1**).

Among the 21 GxAlc interaction loci, most were identified by the EUR, EAS, or CPMA analyses, which have the largest sample sizes and thus the highest power to detect GxAlc interactions (**Supplementary Figure 1**). Two loci were identified only through AFR meta-analyses (*DHFR* and *ESRRG*), and four only through HIS meta-analyses (*RLF, DNM3, C8orf37-AS1*, and *OBP2B*). Of the 21 loci, 10 interactions were population-specific. Four were population-specific because the variants could only be analyzed in one population (*CACHD1* and SIX2 in EUR, and *GRM8* and *BRAP* in EAS, which were too rare in other populations), and 6 were population-specific due to significant heterogeneity of interaction effects between populations (*DNM3*, *ESRRG*, *CACNA1D*, *DHFR*, *OBP2B*,, and *TLN2;* P_het_<0.05/17, corrected for 17 variants analyzed in more than one population, **Supplementary Figure 2**). Seven loci demonstrated significant between-sex heterogeneity (P_sexhet_ < 0.05/21; *PINK1*, *RLF*, *ESRRG*, *FGF5*, *C8orf37*, *OBP2B*, and *RPH3A*), and 2 loci were present only in males (*DHFR* and *ANXA2*). The *ESRRG* encodes the EstrogenRelated Receptor Gamma. Most GxAlc interaction loci were identified for the outcome PP (12 loci, compared to 3 for DBP, and 9 for SBP) and for the exposures LIGHTvsNEVER and HEAVYvsNEVER (7 loci each, compared to 5 for CURDRINK and HEAVYvsLIGHT each, **Supplementary Figure 1, Supplementary Table 3**).

**Figure 3.**
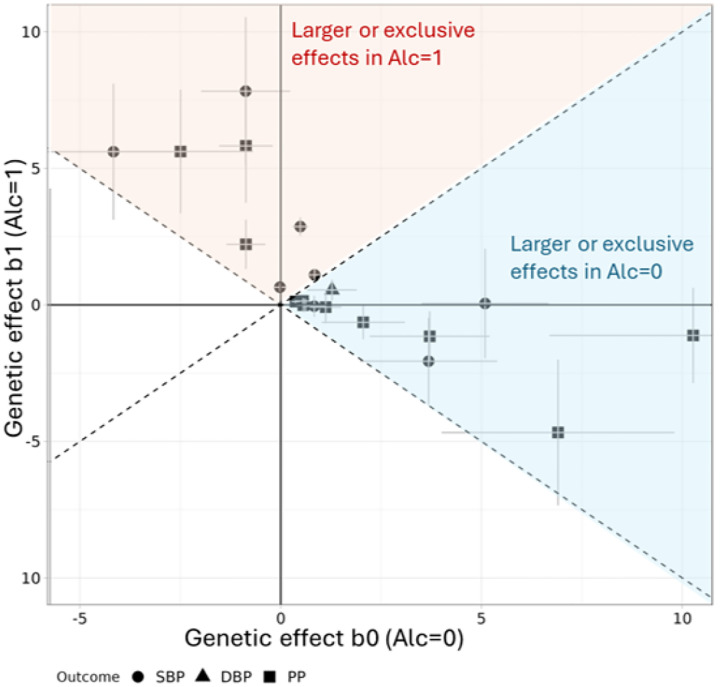
Gene-alcohol interaction at 21 interaction loci on BP. The estimated genetic effect sizes on BP in the respective unexposed (b_0_, X axis) and exposed (b_1_, Y axis) alcohol drinking subgroups are shown. The unexposed effect sizes are equivalent to the estimated genetic main effect from the 2df model, and the exposed effect sizes were obtained from the main and interaction effects. Estimates are provided for the 21 interaction variants in the respective identifying analysis (i.e., the population/sex/BP/Alc combination in which the variant showed the smallest P_GxA__lc_; **Table 1).**

When comparing the genetic effects on BP between exposed and unexposed individuals, we found that increased alcohol consumption differentially affected mostly decreased genetic effects on BP. Thirteen loci had larger or exclusive effects on BP in unexposed individuals, compared to 8 that had larger or exclusive effects in exposed individuals (Figure 3).

In summary, our extensive GWIS identified 21 significant GxAlc interactions across multiple BP traits and population samples, with variation in the direction of the GxAlc interactions.

### GWIS meta-analyses from the 2df test identify 25 additional GxAlc interaction BP loci

A total of 701 unique genomic regions (distance >500kb) were identified through our 2df joint main and interaction meta-analyses, including 2,106 independent loci (r^2^<0.1) with genome-wide significant 2df joint effects for BP traits (P_2df_<5×10^−8^, FDR_2df_<5%, **Supplementary Table 4**, Figure 1). Among these 2,106 loci, we identified 30 secondary GxAlc interaction loci (Approach *C*; P_GxAlc_<0.05/M_2df_, Bonferroni-corrected for the population-specific number of 2df loci; **Table 2, Supplementary Figure 3**). Notably, 5 of these 30 secondary GxAlc interaction loci also overlapped with 21 primary GxAlc interaction loci from 1df tests (**Table 1**), while the remaining 25 secondary GxAlc interactions were additionally detected from 2df joint main and interaction effects (**Table 2**). Thus, we identified 46 GxAlc interaction loci, including 21 primary and 25 secondary loci. Among these 46 loci, 7 were located at novel BP loci (three primary, **Table 1**; four additional secondary, **Table 2**).

### GWIS meta-analyses identify 30 novel significant BP loci without gene-alcohol interaction

Finally, we identified 30 genome-wide significant 2df joint loci for BP traits without GxAlc interaction (distance >500kb or r^2^<0.1 between loci; P_2df_<5×10^−8^, FDR_2df_<5%, and P_GxAlc_>0.05/ M_2df_; **Table 3**). Most of these novel BP loci were identified in the CPMA (n=28) and EUR (n=1) analyses, which included large datasets, whereas only one locus was found in the smaller-sample HIS population. Notably, 21 of these 30 loci were identified as significant in a standard main-effect-only GWAS model in CPMA (P_Marginal_<5×10^−8^), suggesting this is likely due to the large sample size and the inclusion of multiple populations.

In summary, the 2df joint meta-analyses identified 36 novel BP loci (6 with and 30 without GxAlc interaction, **Tables 2 and 3**). Although both the 36 novel and 2,070 known BP loci were significantly enriched for nominally significant GxAlc interactions (P_enrich_=9.5×10^−9^ and 4.7×10^−40^, respectively), the proportion of nominally significant interactions (P<0.05) was higher among the novel loci compared to the known ones (13 of 36 =36%, **Table 3**; versus 258 of 2,070 =12%, **Supplementary Table 4**). This demonstrates that considering GxAlc interactions through 2df joint meta-analyses, particularly, helps uncover novel BP associations that might have been missed in the main-effect-only GWAS model.

### Identified BP loci are linked to genes related to alcohol consumption and BP regulation

To determine whether the identified BP loci include genes previously associated with alcohol consumption and BP, we accessed the NHGRI-EBI GWAS catalog (see Methods). We also examined the literature on human studies and animal experimental models.

We initially analyzed 46 significant GxAlc interaction loci, including 21 primary and 25 secondary GxAlc interactions. According to the GWAS catalog, several genes at these BP loci are associated with alcohol consumption, alcohol dependence, or AUD (e.g., *SIX2, LINC01833, GRM8, ANXA2, GRIK2, DNM3, ESRRG, FIGN, CHDH, ADH1A, ADH1B, ADH1C, RASGRF2, ALDH2, HECTD4, BRAP, RPH3A, COBLL1, TMEM161B*, *MIR9–2HG, UQCRQ, BCL7B, PPP1R3B-DT, GARIN2, FURIN, FTO, WNT3*, among many others, **Supplementary Table 5, Supplementary Table 6**). Notably, two loci (4q23 and 12q24.11-q24.13), known to be associated with BP traits, harbor key genes involved in alcohol metabolism. The 4q23 locus contains genes encoding isoenzymes of alcohol dehydrogenase, which primarily catalyze alcohol oxidation. The class I enzymes (*ADH1A, ADH1B, and ADH1C*), responsible for most liver alcohol metabolism, are characterized by high abundance and low affinity for ethanol. The 12q24.12-q24.13 locus harbors the *ALDH2* gene, which encodes an enzyme that metabolizes acetaldehyde, a toxic byproduct of alcohol metabolism. The ADH1B*2 and ALDH2*2 alleles, common in Eastern Asian populations, alter alcohol metabolism: ADH1B*2 accelerates the conversion of alcohol to toxic acetaldehyde [29], while ALDH2*2 impairs the detoxification of acetaldehyde [29, 30]. Furthermore, studies in humans and animals have demonstrated that several GxAlc interaction loci for BP harbor genes associated with alcohol dependence, AUD, and HTN (**Supplementary Table 5**). These findings suggest that modeling GxAlc interactions in GWIS meta-analyses helps identify genetic variants that influence BP through alcohol metabolism and provides insights into the mechanisms behind alcohol-related BP levels and HTN.

Next, we examined the 30 novel BP loci without GxAlc interaction and found that some of these had been previously reported to have suggestive associations (4×10^−8^<P<1×10^−5^) with BP traits or HTN in the GWAS catalog (e.g., *SHISAL2A, LINC02147, C6orf141-GLYATL3, TYW1B, LINC03021/CSMD1, ARHGAP22, LOC105369874*, and *LINC01643*), as briefly described in **Supplementary Table 6**. Additionally, several genes in these novel BP loci have been linked to BP regulation in human and animal studies (**Supplementary Table 6**). Therefore, the large sample sizes in these populations, especially in CPMA, increased statistical power, enabling these loci to reach genome-wide significance.

In summary, several GxAlc interaction loci were linked to genes related to alcohol consumption and BP regulation, while novel BP loci without GxAlc were associated with genes involved in BP regulation.

### Druggability assessment at 46 GxAlc interaction loci

We examined the potential druggability of genes located at the 46 GxAlc interaction loci (see Methods), as previously described [31]. We first queried the top-prioritized mapped gene targets using the Drug-Gene Interaction database (DGIdb), which identified 16 genes annotated as clinically actionable or part of the druggable genome (**Supplementary Table 7**). These include gene targets involved in GPCR signaling (*PDE10A, RASGRF2,* and *GRM8*) or tryptophan metabolism (*ALDH2*). We found 7 gene targets of approved drugs evaluated in late-stage clinical trials through the DrugBank and ClinicalTrials databases (**Supplementary Table 8**). Notably, we identified *IL17RB*, a target of the monoclonal antibody brodalumab, which is approved for the treatment of moderate-to-severe plaque psoriasis. Previous studies also linked genetic variants in *IL17RB* and IL17RB protein levels to responses to acamprosate, a drug approved for the treatment of AUD [32]. We identified *PDE10A* as a target of cyclic nucleotide phosphodiesterase inhibitors, such as dipyridamole, approved to prevent transient ischemic attack and ischemic cerebral infarction, and papaverine, approved for the treatment of vascular spasms [33]. Additionally, we identified two glutamic acid targets (*GRIK2* and *GRM8*). An experimental vaccine (rhGAD65) designed to block the action of autoantibodies to glutamic acid decarboxylase has been explored as a therapy for Latent Autoimmune Disease in Adults [34], and drugs that inhibit glutamate receptors, such as amantadine and safinamide, can reduce motor complications in patients with Parkinson’s disease [35]. Lastly, we identified *DBH* as a target of the established drug, disulfiram, used to treat alcohol and narcotic drug addiction [36, 37].

### Variant annotation highlights GxAlc interaction variants with functional and regulatory relevance and their involvement in ethanol oxidation and fatty acid metabolism

We then analyzed whether functionally or regulatory relevant variants were tagged at the 46 GxAlc interaction loci and whether the mapped genes were enriched for specific biological and molecular pathways (see Methods). We annotated the 46 lead variants and their proxies (r^2^>0.6) using FUMA SNP2GENE to assess their functional relevance and effects on gene expression in relevant GTEx tissues (adrenal gland, brain, arteries, heart, liver, and kidney). This analysis identified four non-synonymous missense variants in *ADH1B, SLC22A4, TNKS,* and *ALDH2* (**Supplementary Table 9**) and numerous significant eQTLs influencing the expression of 40 genes in at least one of the queried GTEx tissues (**Supplementary Table 10**). We then performed a FUMA GENE2FUNC analysis of the mapped genes, which uncovered several significantly enriched gene sets (FDR<5%) primarily involved in ethanol oxidation and fatty acid metabolism (**Supplementary Table 11**).

### Aggregated evidence and gene prioritization at the 46 GxAlc interaction loci

**Figure 4.**
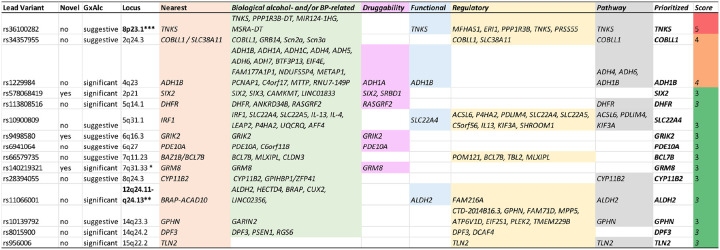
Gene prioritization among 46 gene-alcohol interaction loci influencing BP levels. The figure highlights 15 loci with 15 prioritized genes identified by at least 3 annotation sources as relevant among the 46 GxAlc interaction loci. Genes were scored based on positional mapping (nearest genes), biological relevance, druggability, the presence of variants with functional and regulatory annotations, and their inclusion in a significantly enriched gene set (pathway). A full summary of all loci and scored genes is provided in **Supplementary Table 12.**

Finally, for the 46 GxAlc interaction loci, we combined 7 lines of evidence for the highlighted genes, including biological insights from literature, druggability, functional and regulatory annotations, nearest gene, and pathways, into a single gene prioritization table (**Supplementary Table 12**). Using this integrated evidence, we identified 15 genes prioritized by at least 3 approaches (gene prioritization score ≥3 across these 7 lines of evidence; Figure 4). Three of these genes were novel BP loci: *SIX2*, *GRM8*, and *GRIK2*, each mapped to the nearest gene and possessing biologically relevant and druggable properties. The top-scoring gene across all GxAlc interaction loci was *TNKS*, which was closest to the lead variant and displayed biologically relevant features, including a missense variant, an eQTL, and an enriched gene set.

## Discussion

This GWIS meta-analysis, including over 1.1 million individuals from multiple populations, is the largest dataset to date for examining how genetic variation interacts with alcohol consumption to influence BP traits. By incorporating population diversity, regional differences in alcohol use, dose-dependent drinking categories, and sex-specific effects, the study increased statistical power to detect GxAlc interactions and identify novel BP loci. We found 46 GxAlc interaction loci significantly associated with BP levels, including 21 from a 1df test and 25 from a 2df joint test of main and GxAlc interaction effects. Among these, seven loci are novel for BP, mapped to *SIX2*, *GRM8*, *GRIK2*, *C7orf33/CUL1*, *LINC01683*, and *LOC105370839/ANXA2* (two independent variants), while the remaining GxAlc interaction loci have been previously reported for BP in GWAS.

Among the 21 significant GxAlc interaction loci, 10 were population-specific. Four of these loci were found only in one population or were missing or filtered out in others due to low frequency, such as the *ALDH2* region in EAS. Six loci displayed significant between-population heterogeneity, like *ESRRG*, which was specific to AFR. The GxAlc interaction loci identified for BP levels may partly reflect differences in sample sizes and genetic diversity across populations. Variations in allele frequencies, LD structures, and gene expression by population could **influence the genetic factors underlying complex traits and diseases** [2, 15–17]. Research indicates that African Americans and other individuals of African ancestry often face a greater burden of alcohol-related health issues, including HTN, and other cardiometabolic diseases, despite generally consuming less alcohol than those of European descent [38, 39]. Furthermore, HTN tends to be more prevalent, occurs earlier, and results in more severe outcomes in populations of African descent than in other groups, especially Europeans. Variations in alcohol consumption and HTN across populations are influenced by a complex interplay of factors, including genetics, increased sodium sensitivity, environmental influences, and socioeconomic status [15, 38, 39].

Genetic variants can have larger effect sizes in some populations than in others. Notable examples include the GxAlc genes, which encode isoenzymes of alcohol dehydrogenase involved in ethanol metabolism and the detoxification of other alcohols and aldehydes. Our findings **provide** evidence that the *ADH1B* missense variant rs1229984 (4q23), along with the *HECTD4* (12q24.13) intronic variant rs11066280 and the *BRAP* intronic variant rs11066001 (12q24.12), interact with alcohol consumption to influence SBP levels in current drinkers. The allele frequencies of rs1229984-T, rs11066280-A, and rs11066001-C vary across populations. These variants are common in East Asian populations but are **uncommon**, rare, or monomorphic in European, African American, and Latin groups, according to NCBI-ALFA (e.g., rs1229984-T: 0.70 in EAS versus T=0.04 in EUR). These genes are known for their associations with alcohol consumption, alcohol dependence (tolerance), sensitivity to alcohol (e.g., flushing), AUD, and BP traits [18, 29, 30, 40–42]. Both *HECTD4*-rs11066280 and *BRAP*-rs11066001 are also correlated with the *ALDH2* missense rs671 (12q24.12, r^2^=0.48 and D’=0.76, and r^2^=0.97 and D’=0.99, respectively, in EAS populations). A study of Taiwanese participants found strong evidence for additive and synergistic risks associated with functionally important *ADH1B* rs1229984 and *ALDH2* rs671 variants for alcohol-related disorders and upper aerodigestive tract cancer [30]. Heavy alcohol consumption increases the risk for esophageal cancer by 381% in individuals carrying the rs1229984 TC/CC and rs671 GA/AA genotypes [30]. Additionally, HTN has been linked to oral, laryngeal, and esophageal cancers in Koreans after adjusting for confounders, including alcohol consumption [43]. The underlying mechanisms remain under investigation; potential causes include chronic inflammation and oxidative stress. A study showed that acetaldehyde induces DNA damage and impairs mitochondrial functionality in rats, generating oxidative stress, which sensitizes hepatocytes to oxidative damage, contributing to the development of alcoholic liver disease [44]. These findings suggest that reducing excessive alcohol consumption, particularly in individuals with high-activity *ADH1B* and slow-acting *ALDH2* variants, which may lead to high buildup of toxic acetaldehyde, could help decrease the risk of HTN, CVD, and certain cancers.

Research involving both human and animal models provides evidence that several novel (e.g., *GRM8, SIX2, ANXA2, GRIK2,* and *CUL1*) and known (e.g., *BCL7B, DPF3, RASGRF2, DHFR, PDE10A, DBH, SLC22A4, SLC22A5, DNAJC10,* and *EOMES*) GxAlc interaction loci associated with BP levels contain genes linked to alcohol dependence, AUD, neurological, and psychiatric conditions, as well as HTN, as summarized in **Supplementary Table 5**. For example, *GRM8* encodes a metabotropic glutamate receptor involved in neurological and psychiatric disorders, including alcohol and cocaine dependence [45, 46], while the ionotropic glutamate receptor encoded by *GRIK2* may increase the risk of developing AUD by altering the rewarding and reinforcing effects of alcohol [47]. Both receptors play critical roles in the autonomic regulation of arterial pressure [48].

A deficiency of the transcription factor *Six2* during prenatal development in mice has been linked to chronic renal failure and high BP [49]. Another mouse study indicated that isoliquiritigenin, a compound with anti-hepatic fibrosis properties, reduced the development of alcoholic liver disease by inhibiting *ANXA2*, which plays a role in the cellular response to oxidative stress [50]. The *CUL1* gene affects how alcohol impacts the body, especially in the liver and muscle tissue. Alcohol exposure can cause *CUL1* protein to accumulate in the nucleus of liver cells, blocking mitophagy and leading to liver damage typical of AUD. Additionally, targeting the *DUSP1/CUL1* pathway might be a promising approach to restore mitophagy and treat alcohol-related liver disease [51] caused by such mechanisms.

The known BP-associated GxAlc interaction genes, *RASGRF2* and *DHFR*, play important roles in alcohol consumption. *RASGRF2* (located at 5q14.1) is essential in neural processes related to the rewarding effects of alcohol, affecting both alcohol-seeking behavior and the brain’s dopamine response to alcohol. It has been proposed as a potential therapeutic target for alcohol-related disorders [52]. *DHFR*, located about 300kb from *RASGRF2* at 5q14.1, encodes a vital enzyme that incorporates dietary folic acid into the reduced folate pool. Although studies have reported a link between heavy alcohol consumption and decreased folate absorption, the specific molecular mechanisms remain unclear [53]. Additionally, a study of *DHFR* knockout mice showed higher BP and the development of abdominal aortic aneurysms when exposed to angiotensin II [54]. This suggests that *DHFR* could be a new target for treating high BP [55] and pulmonary HTN [54].

The *DNAJC10* gene encodes the endoplasmic reticulum (ER)-resident chaperone protein ERdj5 [56]. Hepatic *Dnajc10* (ERdj5) mRNA levels increased in both human and mouse cases of alcoholic hepatitis [57]. The mouse study showed that alcohol-induced ERdj5 can regulate the Nrf2 pathway and glutathione levels, offering protective effects against liver damage and oxidative stress caused by alcohol [57].

Another prominent gene is *DBH*, which catalyzes the conversion of dopamine to noradrenaline, a potent vasoconstrictor. *DBH* may also influence neurotransmitter function and various psychiatric traits, including alcohol dependence [37]. Variants of the *DBH* gene are significantly associated with female alcohol dependence and are linked to a higher risk of depression in alcohol-dependent patients [36]. Two of our priority genes, *TNKS* and *COBLL1*, are involved in the *MIR3128*-enriched gene set and are expressed in the brain, among other tissues; however, there is no well-established, direct connection between these genes, alcohol consumption, and HTN in the literature.

Identifying significant GxAlc interaction effects on BP greatly expands the list of variants where alcohol’s HTN impact is either amplified or reduced. This provides biological insights into alcohol-metabolizing pathways beyond the well-known EAS *ALDH2/ADH1B* loci. Many of these variants vary in allele frequency across populations, which might help explain some of the differences in alcohol-related HTN risk. Incorporating these interactions in polygenic scores could greatly improve personalized predictions of alcohol-induced BP increases.

Despite several strengths, such as large sample sizes across multiple populations, extensive efforts to standardize alcohol exposure, and the use of complementary methods to identify statistical interactions, several limitations should be acknowledged. First, the observed GxAlc interactions could be confounded by interactions between genetic variants and unmeasured factors associated with alcohol consumption, or by interactions between alcohol and unmeasured factors associated with the genetic variants themselves [58]. However, this confounding is likely minimized by including covariate-by-alcohol interactions in our models. Nonetheless, the enrichment of alcohol-related genes among the identified GxAlc loci supports the idea that most of these interactions are genuinely related to alcohol exposure. Second, we cannot entirely rule out reverse causality in the data analyzed here, which may emerge from adopting alcohol consumption because of high BP [59]. A more comprehensive assessment of reverse causation would involve incorporating longitudinal data into GxAlc interaction models and properly accounting for changes over time in BP or alcohol consumption. Third, when causal variants for BP and alcohol consumption are located close together, complex LD patterns may produce spurious GxAlc interaction signals [60]. Fourth, because the 1df GxAlc interaction depends statistically on the 2df joint main and interaction, our findings of secondary GxAlc interactions might be overestimated; therefore, the secondary GxAlc results from 2df (25 loci) may require further validation. Despite these limitations, several novel and known loci from GxAlc interactions related to BP traits are associated with genes involved in alcohol consumption, alcohol dependence, AUD, neurological, and psychiatric conditions, as well as HTN. Finally, our study sample primarily comprised individuals from European and East Asian populations, underscoring the need for larger and more diverse cohorts. Addressing these challenges will require biobank-scale datasets that integrate dense variant sets, detailed environmental exposure data, and longitudinal phenotypic measurements across diverse populations.

In summary, the GWIS statistical method enabled us to identify 46 GxAlc loci (including 7 novel ones) that influence BP levels. These findings provide evidence that different genes interacting with alcohol consumption regulate BP and are part of complex networks of molecular mechanisms involved in developmental biology and organ dysfunction. These interactions regulate cellular communication, allowing cells to sense and respond to alcohol exposure, which ultimately causes BP variation and HTN. Their roles extend across biology, affecting key physiological processes and being essential for understanding the mechanisms behind alcohol-induced high BP. These results may lead to potential therapeutic targets for managing BP levels and preventing further heart disease, stroke, kidney disease, peripheral artery disease, alcoholic cardiomyopathy, cirrhosis, and AUD.

## DATA and METHODS

This study is part of the Cohorts for Heart and Aging Research in Genomic Epidemiology (CHARGE) consortium Gene-Lifestyle Interactions working group [61]. Participants in the study were 18 years or older and provided written informed consent. Each participating study was approved by its respective research ethics committees and/or institutional review boards (**Supplementary Material**) and adhered to the principles outlined in the Declaration of Helsinki.

### Study cohorts, phenotype, and alcohol consumption

A total of 1,151,199 individuals from 70 studies, including 111 unique study-population groups from African (AFR, 19 cohorts, N=78,252), Brazilian (BRA, 1 cohort, N=1,748), East Asian (EAS, 15 cohorts, N=177,153), European (EUR, 64 cohorts, N=843,627), Hispanic/Latino (HIS, 9 cohorts, N=37,779), and South Asian (SAS, 3 cohorts, N=12,640) populations based on self-reported ancestry (**Supplementary Table 1**). Descriptions of each participating cohort and their acknowledgments and funding are provided in the **Supplementary Material**.

We analyzed three BP traits. Systolic BP (SBP, in mmHg) and diastolic BP (DBP, in mmHg) were measured either in the resting or sitting position by averaging up to three BP readings taken during the same clinical visit. To account for reductions in BP levels caused by antihypertensive medications, BP readings were adjusted by adding 15 mm Hg to SBP and 10 mm Hg to DBP [16]. After adjustment, pulse pressure (PP) was calculated as the difference between SBP and DBP. Extreme BP values were winsorized if any measurement exceeded 6 SD from the mean, with the value set to exactly 6 SD from the mean.

Alcohol consumption was defined using the US standard drink (StDrk), according to the National Institute on Alcohol Abuse and Alcoholism [62]. One US StDrk contains about 14 grams (0.6 fl oz) of pure alcohol, which is equivalent to the Standard Volume (SV) and Alcohol by Volume (% ABV) listed on the bottle’s label. Examples of US StDrk include a 12 fl oz bottle or can of beer with approximately 5% alcohol, a 5 fl oz glass of wine at about 12% alcohol, or a standard 1.5 fl oz shot of 80-proof spirits such as gin, vodka, or whiskey at roughly 40% alcohol. To standardize alcohol consumption across studies, if needed, we provided 3 options to estimate the approximate US StDrk based on SV and ABV: (a) convert any beverage volume (ml or fl oz) to SV in fl oz (as cited above); (b) use the conversion of country-specific StDrk from grams to US StDrk, which is 14 grams of pure alcohol [63]; or (c) for countries without a StDrk definition, calculate the weight (in grams) of pure alcohol (ethanol) by multiplying the beverage volume (in ml) by the % ABV, then multiplying by 0.78945 g/ml (the ethanol density in 100 ml of solution at 20°C), and finally dividing by 14 grams of pure alcohol.

Participants self-reported their alcohol consumption as drinks per week (DPW) and were categorized into four exposure groups: (1) CURDRINK: current drinker =1 versus not a current drinker =0; (2) LIGHTvsNEVER: light drinkers =1 (1–7 DPW for females or 1–14 DPW for males) versus never drinkers =0 (<1 DPW); (3) HEAVYvsNEVER: heavy drinkers =1 (>7 DPW for females or >14 DPW for males) versus never drinkers =0; and (4) HEAVYvsLIGHT: heavy drinkers =1 versus light drinkers =0. Very heavy drinkers (DPW ≥6 SD from the mean in sex-specific and sex-combined) were excluded from each cohort. The thresholds of heavy drinkers, for females (>7 DPW) and males (>14 DPW), were based on risky alcohol use, as defined by the National Institute on Alcohol Abuse and Alcoholism (NIAAA) [10].

### Genotyping

Genotype imputation was mainly conducted using reference panels from the Trans-Omics for Precision Medicine (TOPMed, https://www.nhlbi.nih.gov/science/trans-omics-precision-medicine-topmed-program) Imputation Server or the Haplotype Reference Consortium (HRC, https://egaarchive.org/studies/EGAS00001001710). Some studies also used imputation based on the ALL ancestry panel from the 1000 Genomes Project (https://www.coriell.org/1/NHGRI/Collections/1000-Genomes-Project-Collection), which utilized haplotypes from 2012–03-14. Variants from autosomal chromosomes and indels (insertions and deletions) were included in the analyses. Details for each cohort regarding genotyping, imputation, and analysis software are provided in **Supplementary Table 2**.

### Genome-wide interaction study (GWIS)

Cohorts performed either linear regression for unrelated individuals or linear mixed-effects models for family data. Two models were used. Model 1 involves the joint analysis of the main and interaction effects: (i) Model 1: *E(Y) = β*_*0*_ *+ β*_*Alc*_ *Alc + β*_*G*_ *G + β*_*GxAlc*_ G*Alc + β_*C*_ *C*. Here, *Y* represents SBP, DBP, or PP; *G* indicates the dosage of the genetic variant; *Alc* denotes alcohol exposure (0/1); *GxAlc* is the variant-alcohol interaction; *β* values are the regression coefficients; and *C* is the matrix of covariates, including age, age^2^, sex (only in combined sex analysis), principal components (PCs), and other study-specific covariates. PCs were derived from genotyped variants in each cohort and used to control for population stratification and genomic confounding. Each cohort determined the number of PCs to be used (**Supplementary Table 2**). The joint model produces cohort-specific estimates of β_G_ and β_GxAlc_, model-based standard errors, and the covariance between β_G_ and β_GxAlc_, as well as P-values from the 2df joint main and GxAlc interaction test (P_2df_) [64, 65]. Additionally, we also evaluated results from a marginal GWAS model: (ii) Model 2: *E(Y) = β*_*0*_ *+ β*_*G*_ *G + β*_*C*_ *C*. Again, *Y* represents the BP traits; *G* the genetic variant; and *C* the matrix of covariates. P marginal-values (P_Marginal_) of Model 1 and Model 2 were applied to each of the 3 BP traits, four alcohol exposures, and sex groups (combined, males, and females) within each population-specific cohort. Association analyses were conducted using MMAP (https://mmap.github.io/), LinGxEScanR (https://github.com/USCbiostats/LinGxEScanR), or GEM (https://github.com/large-scale-gxe-methods/GEM/releases). Meta-analyses were conducted within and across populations by pooling population-specific meta-analysis results.

### Quality control

We implemented strict quality control (QC) procedures at both the cohort association analysis and meta-analysis stages, using EasyQC [66]. Any cohort-specific analytical issues that arose were resolved before conducting the meta-analyses. Variants were filtered out with an imputation quality <0.5 (IMPUTE2: https://mathgen.stats.ox.ac.uk/impute/impute_v2.html or MACH https://bioinformaticshome.com/tools/imputation/descriptions/MaCH.html#gsc.tab=0). Additionally, within each study, variants were filtered based on degrees of freedom, calculated as minor allele count × imputation quality (MAC*R2)<20 for the alcohol-exposed (E=1), unexposed (E=0), and total study samples. A study was excluded from the meta-analysis if it had <100 individuals or <50 individuals in either the alcohol-exposed or unexposed groups. To address genomic inflation (λ) potentially caused by population stratification across studies or by unaccounted relatedness, we applied cohort-specific population-genomic control (GC) corrections. As a result, the λ_GC_ values were approximately 1.0, indicating effective control of false-positive signals.

### Meta-analyses

All meta-analyses were conducted using METAL [24]. From GWIS Model 1, we performed both (1) 1df inverse-variance weighted meta-analyses of GxAlc interaction effects [23]and 2df joint main and interaction meta-analyses [24]. Additionally, we performed inverse-variance-weighted 1df meta-analyses of marginal *G* effects (main-effect-only GWAS) for Model 2 [24]. Single-stage GWIS and GWAS meta-analyses of BP traits were carried out within each population-specific group (AFR, EAS, EUR, HIS, and SAS). Summary meta-analyses of significant association results within specific populations were reported if they included >5,000 individuals in AFR, HIS, and SAS, or, due to larger sample sizes in EUR and EAS, >20,000 in EUR and EAS, with at least two studies contributing for each variant. Finally, we meta-analyzed GWIS and GWAS summary statistics across all populations for each alcohol-BP trait combination. Cross-population meta-analysis results were reported when the total sample size exceeded 20,000 and at least 2 populations contributed; thus, variants that did not meet population-specific criteria might still be included in the cross-population results due to the larger overall sample size.

For targeted interaction search, variants with significant GxAlc interaction were selected based on the 1df interaction meta-analysis using two approaches: (i) considering genome-wide significance on the GxAlc interaction (P_GxAlc_<5×10^−8^ and FDR<5%, Approach *A*), and (ii) a 2-step approach that involves filtering for marginal association (P<10^−5^) and testing the filtered variants for GxAlc interaction at a reduced Bonferroni-corrected alpha level in the second step (P_GxAlc_<0.05/M_eff_, and FDR<5%; where M_eff_ is the number of effectively independent tests among all filtered variants, estimated based on PCA; Approach *B*; separately by population, sex, outcome, and exposure) [67]. For the 2df analysis results, variants were selected based on 2df joint meta-analysis and genome-wide significance (P_2df_<5×10^−8^ and FDR<5%). For variants with significant joint effects, P_GxAlc_ was also evaluated to determine whether the 2df result reflected an interaction or was mainly driven by the main effect of the variant. Associations were labeled as secondary interactions if they met a Bonferroni-corrected GxAlc alpha level (^P^_GxAlc_<0.05^/M^_2df_^, where M^_2df_ is the number of 2df joint effect variants; Approach *C*; determined separately by population). In addition, the 2df analysis results were reported for the main effect only (without GxAlc interaction, Approach *D*: P_2df_<5×10^−8^, FDR_2df_<5%, and P_GxAlc_>0.05/M_2df_). The locus definition refers to the lead variant showing the most significant interaction/joint effect after applying LD-based clumping (r^2^≥0.1 for all variants of the locus). Variants missing in the LD reference panel (ancestry-specific TOPMed-imputed 1000G panels) but located within genomic regions not identified by the clumping (distance>500kb) were retained as independent loci. A novel locus for BP was identified if all locus variants were more than ±500 kb away from previously reported (known) BP genetic associations in the NHGRI-EBI GWAS catalog (mapped to Genome Assembly GRCh38.p14 and dbSNP Build 156; https://www.ebi.ac.uk/gwas/), or if all locus variants and known BP variants were in linkage equilibrium (*i.e*., not correlated, r^2^<0.1).

For comparisons of allele frequencies among populations, the Allele Frequency Aggregator (ALFA) from the National Center for Biotechnology Information (NCBI; www.ncbi.nlm.nih.gov/snp) was used. NCBI was also assessed to report the functional consequences of variants.

### Heterogeneity by sex

We examined sex-specific heterogeneity by analyzing sex-specific beta coefficients of the GxAlc interaction. Evidence of heterogeneity between sexes was evaluated using two-sample Z-tests, assuming independence between males and females, which is a conservative approach [68]. Significant sex heterogeneity was identified at the Bonferroni-adjusted significance level.

### Variant annotation and gene-set enrichment analysis

To annotate identified GxAlc variants for their functional and regulatory effects, we performed FUMA SNP2GENE analysis (https://fuma.ctglab.nl/) [69]. We included all identified GxAlc lead variants and their proxies (r^2^>0.6) in the annotation. Based on FUMA Annovar annotation, we selected protein-altering missense variants with non-synonymous, stop-loss, or stop-gained consequences. Significant eQTLs were chosen at FDR<5% using relevant GTEx tissues and cell types (adrenal gland, brain, arteries, heart, liver, and kidney; https://gtexportal.org/home/). Mapped genes were analyzed using the FUMA GENE2FUNC gene set enrichment analysis, and significantly enriched gene sets were selected with FDR<5%.

### Druggability analysis

We initially used the Drug-Gene Interaction database [31] (DGIdb, v5.0; https://dgidb.org/) to identify high-priority genes in 46 GxAlc interaction loci to assess the druggability of candidate gene targets. We annotated genes for their involvement in pathways and functions using the Kyoto Encyclopedia of Genes and Genomes (KEGG, https://www.genome.jp/kegg/) database. We annotated the druggability target categories and queried all interacting drugs reported in 47 data sources, including BaderLabGenes, CarisMolecularIntelligence, dGene, FoundationOneGenes, GO, HingoraniCasas, HopkinsGroom, HumanProteinAtlas, IDG, MskImpact, Oncomine, Pharos, RussLampel, Tempus, CGI, CKB-CORE, CIViC, COSMIC, CancerCommons, ChemblDrugs, ChemIDPlus, ChemblInteractions, ClearityFoundationBiomarkers, ClearityFoundationClinicalTrial, DTC, DoCM, DrugBank, Drugs@FDA, Ensembl, HGNC, NCBI, FDA, GuideToPharmacology, HemOnc, JACX-CKB, MyCancerGenome, MyCancerGenomeClinicalTrial, NCI, NCIt, OncoKB, PharmGKB, RxNorm, TALC, TEND, TTD, TdgClinicalTrial, and Wikidata. We also queried protein targets for available active ligands in ChEMBL v36. Gene targets within the druggable genome were identified using the latest list from the NIH Illuminating the Druggable Genome Project, accessible on the Pharos platform (https://github.com/druggablegenome/IDGTargets). Additionally, we examined FDA-approved drugs, late-stage clinical trials, and disease indications through DrugBank v6.06 (https://go.drugbank.com/), ChEMBL v36 [70], and ClinicalTrials.gov (Sept 25, 2025 release, https://clinicaltrials.gov/), highlighting the most relevant MeSH and DrugBank indications and clinical trial data.

## Data and resource availability

All summary results will be available in the GWAS Catalog. Source code for primary software used to conduct meta-analysis is publicly available at the following repositories: GEM (https://github.com/large-scale-gxe-methods/GEM), MMAP (https://mmap.github.io/), LinGxEScanR (https://github.com/USCbiostats/LinGxEScanR), FUMA (https://fuma.ctglab.nl/), METAL (https://github.com/statgen/METAL).

## Supplementary Material

Supplementary Files

This is a list of supplementary files associated with this preprint. Click to download.

- SupplementaryMateriallegends.docx
- SupplementaryTable1.xlsx
- SupplementaryTable10.xlsx
- SupplementaryTable3.xlsx
- SupplementaryTable5.xlsx
- SupplementaryTable12.xlsx
- SupplementaryTable4.xlsx
- Table3.docx
- SupplementaryFigure1.docx
- Table1.docx
- SupplementaryFigure2.docx
- SupplementaryTable7.xlsx
- SupplementaryTable9.xlsx
- SupplementaryMaterial.docx
- SupplementaryTable8.xlsx
- SupplementaryFigure3.docx
- SupplementaryTable6.xlsx
- SupplementaryTable2.xlsx
- Table2.docx
- SupplementaryTable11.xlsx

Tables are available in the Supplementary Files section.

## References

[1] Biddinger K.J., , Association of Habitual Alcohol Intake With Risk of Cardiovascular Disease. JAMA Netw Open, 2022. 5(3): p. e223849. https://pmc.ncbi.nlm.nih.gov/articles/PMC8956974 35333364 10.1001/jamanetworkopen.2022.3849 PMC8956974

[2] Cecchini M., , Alcohol Intake and Risk of Hypertension: A Systematic Review and Dose-Response Meta-Analysis of Nonexperimental Cohort Studies. Hypertension, 2024. 81(8): p. 1701–1715. https://pmc.ncbi.nlm.nih.gov/articles/PMC11251509 38864208 10.1161/HYPERTENSIONAHA.124.22703 PMC11251509

[3] McEvoy J.W., , 2024 ESC Guidelines for the management of elevated blood pressure and hypertension. Eur Heart J, 2024. 45(38): p. 3912–4018. https://pubmed.ncbi.nlm.nih.gov/39210715 39210715 10.1093/eurheartj/ehae178

[4] Roerecke M., , The effect of a reduction in alcohol consumption on blood pressure: a systematic review and meta-analysis. Lancet Public Health, 2017. 2(2): p. e108–e120. https://pmc.ncbi.nlm.nih.gov/articles/PMC6118407 29253389 10.1016/S2468-2667(17)30003-8 PMC6118407

[5] Roerecke M., , Sex-Specific Associations Between Alcohol Consumption and Incidence of Hypertension: A Systematic Review and Meta-Analysis of Cohort Studies. J Am Heart Assoc, 2018. 7(13). https://pmc.ncbi.nlm.nih.gov/articles/PMC6064910 10.1161/JAHA.117.008202 PMC6064910 29950485

[6] Jones D.W., , 2025 AHA/ACC/AANP/AAPA/ABC/ACCP/ACPM/AGS/AMA/ASPC/NMA/PCNA/SGIM Guideline for the Prevention, Detection, Evaluation, and Management of High Blood Pressure in Adults: A Report of the American College of Cardiology/American Heart Association Joint Committee on Clinical Practice Guidelines. J Am Coll Cardiol, 2025. 86(18): p. 1567–1678. https://pubmed.ncbi.nlm.nih.gov/40815242 40815242 10.1016/j.jacc.2025.05.007

[7] Lee H., , Very High Prevalence of Nonoptimally Controlled Traditional Risk Factors at the Onset of Cardiovascular Disease. J Am Coll Cardiol, 2025. 86(14): p. 1017–1029. https://pubmed.ncbi.nlm.nih.gov/41033739 41033739 10.1016/j.jacc.2025.07.014 PMC13519508

[8] CDC Foundation. CDC Foundation Announces New National Hypertension Control Program to Combat Uncontrolled Hypertension. 2025 [2025 Dec 3]; Available from: https://www.cdcfoundation.org/pr/2025/New-Hypertension-Control-Program.

[9] World Health Organization, Global status report on alcohol and health and treatment of substance use disorders. 2024: Geneva, Switzerland.

[10] Patel A.K. and Balasanova A.A., Unhealthy Alcohol Use. JAMA, 2021. 326(2): p. 196. https://pubmed.ncbi.nlm.nih.gov/34255006 34255006 10.1001/jama.2020.2015

[11] Edwards A.C., , Socioeconomic position indicators and risk of alcohol-related medical conditions: A national cohort study from Sweden. PLoS Med, 2024. 21(3): p. e1004359. https://pmc.ncbi.nlm.nih.gov/articles/PMC10950249 38502640 10.1371/journal.pmed.1004359 PMC10950249

[12] Edwards A.C., , Genetic and environmental influences on the progression from alcohol use disorder to alcohol-related medical conditions. Alcohol Clin Exp Res, 2021. 45(12): p. 2528–2535. https://pmc.ncbi.nlm.nih.gov/articles/PMC8712390 34923650 10.1111/acer.14731 PMC8712390

[13] Padovan J.C., , Reactive Oxygen Species Are Central Mediators of Vascular Dysfunction and Hypertension Induced by Ethanol Consumption. Antioxidants (Basel), 2023. 12(10). https://pmc.ncbi.nlm.nih.gov/articles/PMC10604002 10.3390/antiox12101813 PMC10604002 37891892

[14] White A.M., Gender Differences in the Epidemiology of Alcohol Use and Related Harms in the United States. Alcohol Res, 2020. 40(2): p. 01. https://pmc.ncbi.nlm.nih.gov/articles/PMC7590834 10.35946/arcr.v40.2.01 PMC7590834 33133878

[15] Jobe M., , Hypertension in Sub-Saharan Africa: Burden, Barriers and Priorities for Improving Treatment Outcomes. Circ Res, 2025. 137(1): p. 106–118. https://pmc.ncbi.nlm.nih.gov/articles/PMC12175831 40536937 10.1161/CIRCRESAHA.124.323889 PMC12175831

[16] Feitosa M.F., , Novel genetic associations for blood pressure identified via gene-alcohol interaction in up to 570K individuals across multiple ancestries. PLoS One, 2018. 13(6): p. e0198166. https://pmc.ncbi.nlm.nih.gov/articles/PMC6005576 29912962 10.1371/journal.pone.0198166 PMC6005576

[17] Abdellaoui A., , 15 years of GWAS discovery: Realizing the promise. Am J Hum Genet, 2023. 110(2): p. 179–194. https://pmc.ncbi.nlm.nih.gov/articles/PMC9943775 36634672 10.1016/j.ajhg.2022.12.011 PMC9943775

[18] Keaton J.M., , Genome-wide analysis in over 1 million individuals of European ancestry yields improved polygenic risk scores for blood pressure traits. Nat Genet, 2024. 56(5): p. 778–791. https://pmc.ncbi.nlm.nih.gov/articles/PMC11096100 38689001 10.1038/s41588-024-01714-w PMC11096100

[19] Alexander M.R., Edwards T.L., and Harrison D.G., GWAS for Defining the Pathogenesis of Hypertension: Have They Delivered? Hypertension, 2025. 82(4): p. 573–582. https://pmc.ncbi.nlm.nih.gov/articles/PMC11922662 39936322 10.1161/HYPERTENSIONAHA.124.23451 PMC11922662

[20] Trofimov M., , Genetic analysis of alcohol use disorder: GWAS of alcohol use disorders identification test (AUDIT) and polygenic risk scores in an east slavic population. Drug Alcohol Depend, 2025. 273: p. 112713. https://pubmed.ncbi.nlm.nih.gov/40440926 40440926 10.1016/j.drugalcdep.2025.112713

[21] Amaral E.H.B., , Uncovering genetic mechanisms associated with harmful use of alcohol in admixed Latin Americans. BMC Genomics, 2025. 26(1): p. 639. https://pmc.ncbi.nlm.nih.gov/articles/PMC12232578 40624472 10.1186/s12864-025-11691-x PMC12232578

[22] Saunders G.R.B., , Genetic diversity fuels gene discovery for tobacco and alcohol use. Nature, 2022. 612(7941): p. 720–724. https://pmc.ncbi.nlm.nih.gov/articles/PMC9771818 36477530 10.1038/s41586-022-05477-4 PMC9771818

[23] Willer C.J., Li Y., and Abecasis G.R., METAL: fast and efficient meta-analysis of genomewide association scans. Bioinformatics, 2010. 26(17): p. 2190–1. https://pmc.ncbi.nlm.nih.gov/articles/PMC2922887 20616382 10.1093/bioinformatics/btq340 PMC2922887

[24] Manning A.K., , Meta-analysis of gene-environment interaction: joint estimation of SNP and SNP x environment regression coefficients. Genet Epidemiol, 2011. 35(1): p. 11–8. https://pmc.ncbi.nlm.nih.gov/articles/PMC3312394 21181894 10.1002/gepi.20546 PMC3312394

[25] Thliveros P., Uckun Kiran E., and Webb C., Microbial biodiesel production by direct methanolysis of oleaginous biomass. Bioresour Technol, 2014. 157: p. 181–7. https://pubmed.ncbi.nlm.nih.gov/24556371 24556371 10.1016/j.biortech.2014.01.111

[26] Edenberg H.J. and McClintick J.N., Alcohol Dehydrogenases, Aldehyde Dehydrogenases, and Alcohol Use Disorders: A Critical Review. Alcohol Clin Exp Res, 2018. 42(12): p. 2281–2297. https://pmc.ncbi.nlm.nih.gov/articles/PMC6286250 30320893 10.1111/acer.13904 PMC6286250

[27] Gao J., , Aldehyde Dehydrogenase 2 as a Therapeutic Target in Oxidative Stress-Related Diseases: Post-Translational Modifications Deserve More Attention. Int J Mol Sci, 2022. 23(5). https://pmc.ncbi.nlm.nih.gov/articles/PMC8910853 10.3390/ijms23052682 PMC8910853 35269824

[28] Chen I.C., , CUX2, BRAP and ALDH2 are associated with metabolic traits in people with excessive alcohol consumption. Sci Rep, 2020. 10(1): p. 18118. https://pmc.ncbi.nlm.nih.gov/articles/PMC7583246 33093602 10.1038/s41598-020-75199-y PMC7583246

[29] Lin C.L., , The Aldehyde Dehydrogenase ALDH2*2 Allele, Associated with Alcohol Drinking Behavior, Dates Back to Prehistoric Times. Biomolecules, 2021. 11(9). https://pmc.ncbi.nlm.nih.gov/articles/PMC8465343 10.3390/biom11091376 PMC8465343 34572589

[30] Chang T.G., , Impacts of ADH1B rs1229984 and ALDH2 rs671 polymorphisms on risks of alcohol-related disorder and cancer. Cancer Med, 2023. 12(1): p. 747–759. https://pmc.ncbi.nlm.nih.gov/articles/PMC9844601 35670037 10.1002/cam4.4920 PMC9844601

[31] Kavousi M., , Multi-ancestry genome-wide study identifies effector genes and druggable pathways for coronary artery calcification. Nat Genet, 2023. 55(10): p. 1651–1664. https://pmc.ncbi.nlm.nih.gov/articles/PMC10601987 37770635 10.1038/s41588-023-01518-4 PMC10601987

[32] Ho M.F., , IL17RB genetic variants are associated with acamprosate treatment response in patients with alcohol use disorder: A proteomics-informed genomics study. Brain Behav Immun, 2024. **120**: p. 304–314. https://pmc.ncbi.nlm.nih.gov/articles/PMC11269006 38852760 10.1016/j.bbi.2024.06.007 PMC11269006

[33] Menniti F.S., Faraci W.S., and Schmidt C.J., Phosphodiesterases in the CNS: targets for drug development. Nat Rev Drug Discov, 2006. 5(8): p. 660–70. https://pubmed.ncbi.nlm.nih.gov/16883304 16883304 10.1038/nrd2058

[34] Hals I.K., , A 1-year pilot study of intralymphatic injections of GAD-alum in individuals with latent autoimmune diabetes in adults (LADA) with signs of high immunity: No safety concerns and resemblance to juvenile type 1 diabetes. Diabetes Obes Metab, 2023. 25(11): p. 3400–3409. https://pubmed.ncbi.nlm.nih.gov/37580967 37580967 10.1111/dom.15239

[35] Oh E., , Efficacy and safety of safinamide in Parkinson’s disease patients with motor fluctuations without levodopa dosage escalation over 18 weeks: KEEP study. J Neural Transm (Vienna), 2025. 132(3): p. 431–441. https://pmc.ncbi.nlm.nih.gov/articles/PMC11870872 39540934 10.1007/s00702-024-02851-6 PMC11870872

[36] Preuss U.W., , Association of functional DBH genetic variants with alcohol dependence risk and related depression and suicide attempt phenotypes: results from a large multicenter association study. Drug Alcohol Depend, 2013. 133(2): p. 459–67. https://pubmed.ncbi.nlm.nih.gov/23906995 23906995 10.1016/j.drugalcdep.2013.07.002

[37] Soderpalm B. and Ericson M., Alcohol and the dopamine system. Int Rev Neurobiol, 2024. 175: p. 21–73. https://pubmed.ncbi.nlm.nih.gov/38555117 38555117 10.1016/bs.irn.2024.02.003

[38] Zapolski T.C., , Less drinking, yet more problems: understanding African American drinking and related problems. Psychol Bull, 2014. 140(1): p. 188–223. https://pmc.ncbi.nlm.nih.gov/articles/PMC3758406 23477449 10.1037/a0032113 PMC3758406

[39] Desalu J.M., Goodhines P.A., and Park A., Racial discrimination and alcohol use and negative drinking consequences among Black Americans: a meta-analytical review. Addiction, 2019. 114(6): p. 957–967. https://pmc.ncbi.nlm.nih.gov/articles/PMC6510626 30714661 10.1111/add.14578 PMC6510626

[40] Kato N., , Meta-analysis of genome-wide association studies identifies common variants associated with blood pressure variation in east Asians. Nat Genet, 2011. 43(6): p. 531–8. https://pmc.ncbi.nlm.nih.gov/articles/PMC3158568 21572416 10.1038/ng.834 PMC3158568

[41] Sun Y., , Genome-wide association study of alcohol dependence in male Han Chinese and cross-ethnic polygenic risk score comparison. Transl Psychiatry, 2019. 9(1): p. 249. https://pmc.ncbi.nlm.nih.gov/articles/PMC6779867 31591379 10.1038/s41398-019-0586-3 PMC6779867

[42] Hatoum A.S., , Multivariate genome-wide association meta-analysis of over 1 million subjects identifies loci underlying multiple substance use disorders. Nat Ment Health, 2023. 1(3): p. 210–223. https://pmc.ncbi.nlm.nih.gov/articles/PMC10217792 37250466 10.1038/s44220-023-00034-y PMC10217792

[43] Seo J.H., , Hypertension is associated with oral, laryngeal, and esophageal cancer: a nationwide population-based study. Sci Rep, 2020. 10(1): p. 10291. https://pmc.ncbi.nlm.nih.gov/articles/PMC7314820 32581314 10.1038/s41598-020-67329-3 PMC7314820

[44] Farfan Labonne B.E., , Acetaldehyde-induced mitochondrial dysfunction sensitizes hepatocytes to oxidative damage. Cell Biol Toxicol, 2009. 25(6): p. 599–609. https://pubmed.ncbi.nlm.nih.gov/19137438 19137438 10.1007/s10565-008-9115-5

[45] Long E.C., , Further Analyses of Genetic Association Between GRM8 and Alcohol Dependence Symptoms Among Young Adults. J Stud Alcohol Drugs, 2015. 76(3): p. 414–8. https://pmc.ncbi.nlm.nih.gov/articles/PMC4440299 25978827 10.15288/jsad.2015.76.414 PMC4440299

[46] Bauer L.O. and Covault J.M., GRM8 genotype is associated with externalizing disorders and greater inter-trial variability in brain activation during a response inhibition task. Clin Neurophysiol, 2020. 131(6): p. 1180–1186. https://pmc.ncbi.nlm.nih.gov/articles/PMC7198333 32299001 10.1016/j.clinph.2020.02.031 PMC7198333

[47] Yang B.Z., , GRIK1 and GABRA2 Variants Have Distinct Effects on the Dose-Related Subjective Response to Intravenous Alcohol in Healthy Social Drinkers. Alcohol Clin Exp Res, 2017. 41(12): p. 2025–2032. https://pmc.ncbi.nlm.nih.gov/articles/PMC5764175 29131352 10.1111/acer.13516 PMC5764175

[48] Tsuchihashi T., , Metabotropic glutamate receptor subtypes involved in cardiovascular regulation in the rostral ventrolateral medulla of rats. Brain Res Bull, 2000. 52(4): p. 279–83. https://pubmed.ncbi.nlm.nih.gov/10856825 10856825 10.1016/s0361-9230(00)00264-1

[49] Fogelgren B., , Deficiency in Six2 during prenatal development is associated with reduced nephron number, chronic renal failure, and hypertension in Br/+ adult mice. Am J Physiol Renal Physiol, 2009. 296(5): p. F1166–78. https://pmc.ncbi.nlm.nih.gov/articles/PMC2681363 19193724 10.1152/ajprenal.90550.2008 PMC2681363

[50] Liu N., , Isoliquiritigenin alleviates the development of alcoholic liver fibrosis by inhibiting ANXA2. Biomed Pharmacother, 2023. 159: p. 114173. https://pubmed.ncbi.nlm.nih.gov/36680814 36680814 10.1016/j.biopha.2022.114173

[51] Li R., , Interaction between dual specificity phosphatase-1 and cullin-1 attenuates alcohol-related liver disease by restoring p62-mediated mitophagy. Int J Biol Sci, 2023. 19(6): p. 1831–1845. https://pmc.ncbi.nlm.nih.gov/articles/PMC10092755 37063418 10.7150/ijbs.81447 PMC10092755

[52] Stacey D., , RASGRF2 regulates alcohol-induced reinforcement by influencing mesolimbic dopamine neuron activity and dopamine release. Proc Natl Acad Sci U S A, 2012. 109(51): p. 21128–33. https://pmc.ncbi.nlm.nih.gov/articles/PMC3529066 23223532 10.1073/pnas.1211844110 PMC3529066

[53] Sharma J. and Krupenko S.A., Folate pathways mediating the effects of ethanol in tumorigenesis. Chem Biol Interact, 2020. 324: p. 109091. https://pmc.ncbi.nlm.nih.gov/articles/PMC7232643 32283069 10.1016/j.cbi.2020.109091 PMC7232643

[54] Murugesan P., , Reversal of Pulmonary Hypertension in a Human-Like Model: Therapeutic Targeting of Endothelial DHFR. Circ Res, 2024. 134(4): p. 351–370. https://pmc.ncbi.nlm.nih.gov/articles/PMC10880947 38299369 10.1161/CIRCRESAHA.123.323090 PMC10880947

[55] Li H., , Novel Treatment of Hypertension by Specifically Targeting E2F for Restoration of Endothelial Dihydrofolate Reductase and eNOS Function Under Oxidative Stress. Hypertension, 2019. 73(1): p. 179–189. https://pmc.ncbi.nlm.nih.gov/articles/PMC6310047 30571557 10.1161/HYPERTENSIONAHA.118.11643 PMC6310047

[56] Diane A., , Role of the DNAJ/HSP40 family in the pathogenesis of insulin resistance and type 2 diabetes. Ageing Res Rev, 2021. 67: p. 101313. https://pubmed.ncbi.nlm.nih.gov/33676026 33676026 10.1016/j.arr.2021.101313

[57] Hong D.G., , Loss of ERdj5 exacerbates oxidative stress in mice with alcoholic liver disease via suppressing Nrf2. Free Radic Biol Med, 2022. 184: p. 42–52. https://pubmed.ncbi.nlm.nih.gov/35390453 35390453 10.1016/j.freeradbiomed.2022.03.027

[58] Keller M.C., Gene x environment interaction studies have not properly controlled for potential confounders: the problem and the (simple) solution. Biol Psychiatry, 2014. 75(1): p. 18–24. https://pmc.ncbi.nlm.nih.gov/articles/PMC3859520 24135711 10.1016/j.biopsych.2013.09.006 PMC3859520

[59] Smith G.D., Mendelian Randomization for Strengthening Causal Inference in Observational Studies: Application to Gene x Environment Interactions. Perspect Psychol Sci, 2010. 5(5): p. 527–45. https://pubmed.ncbi.nlm.nih.gov/26162196 26162196 10.1177/1745691610383505

[60] Dudbridge F. and Fletcher O., Gene-environment dependence creates spurious gene-environment interaction. Am J Hum Genet, 2014. 95(3): p. 301–7. https://pmc.ncbi.nlm.nih.gov/articles/PMC4157149 25152454 10.1016/j.ajhg.2014.07.014 PMC4157149

[61] Rao D.C., , Multiancestry Study of Gene-Lifestyle Interactions for Cardiovascular Traits in 610 475 Individuals From 124 Cohorts: Design and Rationale. Circ Cardiovasc Genet, 2017. 10(3). https://pmc.ncbi.nlm.nih.gov/articles/PMC5476223 10.1161/CIRCGENETICS.116.001649 PMC5476223 28620071

[62] National Institute on Alcohol Abuse and Alcoholism. What Is A Standard Drink? Acessed [2025 Dec 3]; Available from: https://www.niaaa.nih.gov/alcohols-effects-health/what-standard-drink.

[63] Kalinowski A. and Humphreys K., Governmental standard drink definitions and low-risk alcohol consumption guidelines in 37 countries. Addiction, 2016. 111(7): p. 1293–8. https://pubmed.ncbi.nlm.nih.gov/27073140 27073140 10.1111/add.13341

[64] Kraft P., , Exploiting gene-environment interaction to detect genetic associations. Hum Hered, 2007. 63(2): p. 111–9. https://pubmed.ncbi.nlm.nih.gov/17283440 17283440 10.1159/000099183

[65] Aschard H., , Genome-wide meta-analysis of joint tests for genetic and gene-environment interaction effects. Hum Hered, 2010. 70(4): p. 292–300. https://pmc.ncbi.nlm.nih.gov/articles/PMC3085519 21293137 10.1159/000323318 PMC3085519

[66] Winkler T.W., , Quality control and conduct of genome-wide association meta-analyses. Nat Protoc, 2014. 9(5): p. 1192–212. https://pmc.ncbi.nlm.nih.gov/articles/PMC4083217 24762786 10.1038/nprot.2014.071 PMC4083217

[67] Kawaguchi E.S., , Improved two-step testing of genome-wide gene-environment interactions. Genet Epidemiol, 2023. 47(2): p. 152–166. https://pmc.ncbi.nlm.nih.gov/articles/PMC9974838 36571162 10.1002/gepi.22509 PMC9974838

[68] Winkler T.W., , EasyStrata: evaluation and visualization of stratified genome-wide association meta-analysis data. Bioinformatics, 2015. 31(2): p. 259–61. https://pmc.ncbi.nlm.nih.gov/articles/PMC4287944 25260699 10.1093/bioinformatics/btu621 PMC4287944

[69] Watanabe K., , Functional mapping and annotation of genetic associations with FUMA. Nat Commun, 2017. 8(1): p. 1826. https://pmc.ncbi.nlm.nih.gov/articles/PMC5705698 29184056 10.1038/s41467-017-01261-5 PMC5705698

[70] Zdrazil B., , The ChEMBL Database in 2023: a drug discovery platform spanning multiple bioactivity data types and time periods. Nucleic Acids Res, 2024. 52(D1): p. D1180–D1192. https://pmc.ncbi.nlm.nih.gov/articles/PMC10767899 37933841 10.1093/nar/gkad1004 PMC10767899

